# miR-942 decreases TRAIL-induced apoptosis through ISG12a downregulation and is regulated by AKT

**DOI:** 10.18632/oncotarget.2067

**Published:** 2014-06-06

**Authors:** Nianli Liu, Chaohui Zuo, Xiaohong Wang, Tianran Chen, Darong Yang, Jing Wang, Haizhen Zhu

**Affiliations:** ^1^ Research Center of Cancer Prevention & Treatment, Translational Medicine Research Center of Liver Cancer, Hunan Provincial Tumor Hospital (Affiliated Tumor Hospital of Xiangya Medical School of Central South University), Changsha, China; ^2^ Department of Molecular Medicine, State Key Laboratory of Chemo/Biosensing and Chemometrics, Hunan University, Changsha, China

**Keywords:** Hepatocellular carcinoma, gastric cancer, TRAIL, ISG12a, miR-942

## Abstract

Tumor necrosis factor-related apoptosis-inducing ligand (TRAIL) is an attractive death ligand in targeted cancer therapy. Many cancer cells are refractory to TRAIL-induced cell death and the mechanisms underlying resistance are unclear. The molecular mechanisms of HCC and gastric cancer cells resistant to TRAIL-induced apoptosis were explored using molecular biological and immunological methods. In vivo experiments were conducted to study the effect of interferon stimulated gene 12a (ISG12a) on human liver cancer xenografts in mice. ISG12a decreases in TRAIL-resistant cancer cells. ISG12a regulates the sensitivity of cancer cells to TRAIL in vitro and in vivo. MicroRNA-942 (miR-942) is inversely correlated with ISG12a expression in cancer cells and tissues. Forced expression of miR-942 in TRAIL-sensitive cells significantly reduces endogenous ISG12a level and changes the TRAIL sensitive phenotype to a resistant one. Knockdown of miR-942 expression in TRAIL-resistant cells restores the expression of ISG12a and sensitizes the cells to TRAIL treatment. AKT control TRAIL resistance of cancer cells through downregulation of ISG12a by miR-942. Downregulation of ISG12a by miR-942 is needed to maintain the TRAIL-resistant phenotype of cancer cells and favors cancer cell survival. MiR-942 may offer a novel drug response marker with important implications in designing new therapeutics for **TRAIL** resistant tumors.

## INTRODUCTION

Hepatocellular carcinoma (HCC) and gastric cancer (GC) are among the most common cancers worldwide [1]. Only a small percentage of patients maintain well response to current treatments. New therapeutic approaches are needed for more effective treatment of these cancers. Sorafenib was recently approved for the treatment of advanced renal cell carcinoma and liver cancer patients [2, 3]. However, it can only improve the survival from 7.9 months to 10.7 months for HCC patients [4].

TRAIL selectively induces the death of cancer cells and spares normal cells, so it is an attractive death ligand in targeted cancer therapy [5-8]. TRAIL-induced apoptosis of cancer cells is carried out by activation of mitochondria-independent and mitochondria-dependent intracellular death signaling pathways [9, 10]. Upon the binding of TRAIL, DR4 or DR5 oligomerizes and forms a death-inducing signaling complex (DISC) by recruiting the adaptor protein FADD together with caspase-8, and FLIP. Caspase-8 is activated and then it activates downstream effectors caspase-3 and caspase-7 through both pathways [11]. For the mitochondria-dependent pathway, caspase-8-mediated cleavage of BID leads to oligomerisation of Bax and Bak, resulting in the release of cytochrome c. Cytochrome c activates caspase-9. Activated caspase-3 and caspase-7 provoke cellular destruction by cleaving several hundred cellular proteins including poly ADP-ribose polymerase (PARP).

Although many human tumor cell lines are sensitive to TRAIL agonist-mediated apoptosis and are being evaluated in clinic trials [12, 13], many tumors including HCC and gastric cancer are still resistant to TRAIL-induced apoptosis. Inherent tumor resistance may be a major barrier to effective TRAIL-targeted therapy.

Several mechanisms of resistance to TRAIL have been proposed in different cell types, including death receptor mutation, overexpression of antiapoptotic proteins, and deficiency of BAX [14-17]. No dominant mechanisms of resistance to TRAIL-induced apoptosis have been identified. Clear information about the resistance mechanisms will guide subsequent studies in patient selection and the development of combination trails to counter drug resistance.

ISG12a is highly induced by type I IFN in many cell types [18]. Inhibition of ISG12a expression prevents the sensitization to etoposide-induced apoptosis [19]. Whether ISG12a is involved in TRAIL sensitivity is unclear.

MicroRNAs (miRNAs) are a small noncoding family of 20-25 nucleotides RNAs that play an important role in the negative regulation of gene expression by base-pairing to complementary sites on the targeted mRNAs. It is necessary to explore whether miRNAs modulate TRAIL sensitivity in cancer cells.

Here we demonstrate that miR-942 is upregulated in TRAIL-resistant cancer cells and decreased in TRAIL-sensitive ones. MiR-942 is inversely correlated with ISG12a expression in cancer tissues and cells. AKT control TRAIL resistance of cancer cells through downregulation of ISG12a by miR-942. Downregulation of ISG12a by miR-942 is needed to maintain the TRAIL-resistant phenotype.

## RESULTS

### The cytotoxic effect of TRAIL on human HCC and GC cells

To better understand liver tumor biology, we have attempted to develop HCC cells using liver tumor tissue samples. We established a novel HCC cell line HLCZ02. HLCZ02 cells were derived from grade II differentiated HCC tissue of a male patient (Supplementary Fig. S1A). The cells express human liver-specific genes and proteins albumin and α1-antitrypsin (Supplementary Fig. S1B, S1C).

**Figure 1:**
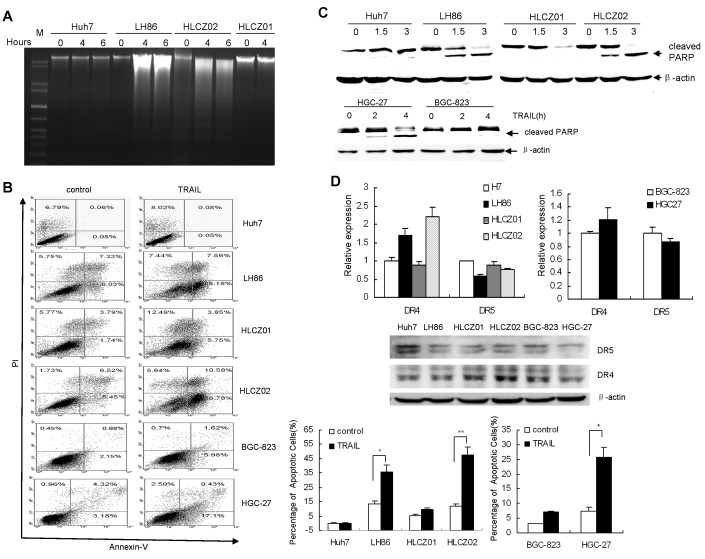
The cytotoxic effect of TRAIL on human HCC and GC cell lines (A) Detection of DNA fragmentation by DNA ladder assay. LH86, Huh7, HLCZ01 and HLCZ02 cells were exposed to TRAIL for 4 hours. 2 μg of cellular DNA was separated on 1% agarose gel at 50V for one hour. The data are one representative of three independent experiments. (B) Detection of apoptosis by flow cytometry and western blot. LH86, Huh7, HLCZ01, HLCZ02, HGC-27 and BGC-823 cells were exposed to TRAIL for 4 hours. Samples were analyzed on a FACS Caliber Cytometer. A minimum of 30000 events per samples were acquired, and subsequently analyzed with CellQuest software. The results are the average of at least three independent experiments. (C) Cleaved PARP was detected by western blot. β–actin was used as control. The data are one representative of three independent experiments. (D) Detection of DR4 and DR5 in HCC and GC cell lines by real-time PCR and western blot analysis. DR4 or DR5 mRNA were detected by real-time RT-PCR and normalized with GAPDH respectively. The results are the average of three independent experiments performed in triplicate. DR4 and DR5 protein was detected by western blot. The data are one representative of three independent experiments.

We examined TRAIL sensitivity of different human HCC cell lines: LH86, Huh7, HLCZ01, HLCZ02 and GC cell lines HGC-27 and BGC-823. Cells were exposed to TRAIL and cell death was tested using DNA ladder assay and flow cytometry. DNA ladder was apparently found in TRAIL-treated LH86 and HLCZ02 cells while rare in Huh7 and HLCZ01 cells (Fig. 1A). Flow cytometry data showed that LH86, HLCZ02 and HGC-27 cells underwent TRAIL-induced apoptosis whereas Huh7, HLCZ01 and BGC-823 cells did not display sensitivity when exposed to soluble TRAIL (Fig. 1B). PARP was cleaved in LH86 and HLCZ02 cells with TRAIL treatment (Fig. 1B). GC cell line HGC-27 was sensitive to TRAIL-induced apoptosis while another GC cell line BGC-823 was resistant to apoptosis (Fig. 1C). A possible mechanism of different sensitivity of tested cells to TRAIL-induced apoptosis could be due to the variable level of the death receptors resulting in increased apoptosis signaling in sensitive cells. However, functional TRAIL receptor isoforms (DR4 and DR5) had comparable level in TRAIL-sensitive cells compared to TRAIL-resistant cells (Fig. 1D). Moreover, all the tested cells had similar expression levels of DcR1 and DcR2 (data not shown).

### TRAIL induces ISG12a in sensitive cells and ISG12a is highly expressed in normal liver tissues and less invasive liver cancer tissues as compared with aggressive liver cancer tissues

**Figure 2:**
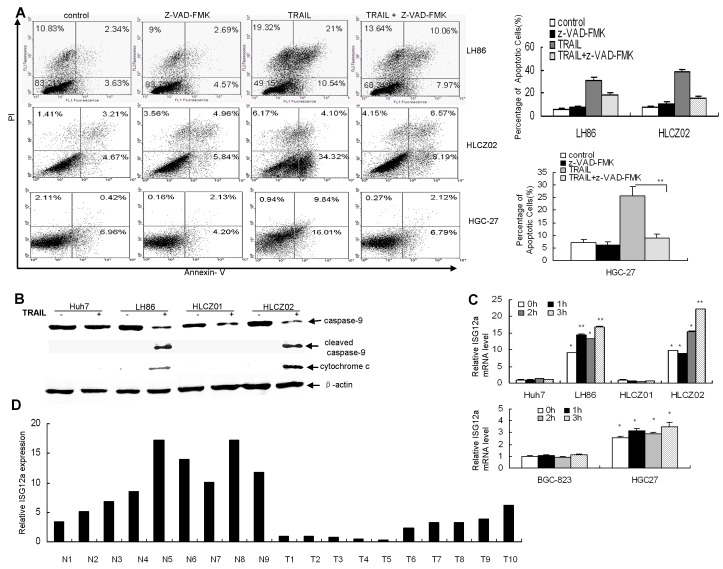
TRAIL induces ISG12a in sensitive cells and ISG12a is highly expressed in normal liver tissues and less invasive liver cancer tissues compared with aggressive liver cancer tissues (A) Pan-caspase inhibitor z-VAD-FMK blocked TRAIL-induced apoptosis of HCC and GC cells. LH86, HLCZ02 and HGC-27 cells were treated by z-VAD-FMK for 2 hours, followed with TRAIL treatment for 4 hours. The cells were analyzed by flow cytometry. The images are one representative of three independent experiments. The results are the average of three independent experiments. (B) Detection of caspase-9 and cytochrome c in TRAIL-treated HCC cells. Huh7, LH86, HLCZ01 and HLCZ02 cells were treated by 10ng/mL of TRAIL for 4 hours. Cleaved caspase-9 and cytochrome c were detected by western blot. A representative result of one from three independent experiments is presented. (C) The time course of ISG12a expression in HCC and GC cells with TRAIL treatment. HLCZ01, Huh7, LH86, HLCZ02, HGC-27 and BGC-823 cells were treated by TRAIL for different time periods. ISG12a mRNA was detected by real-time PCR and normalized with GAPDH. The results are the average of 3 independent experiments performed in triplicate. *P<0.05, **P<0.01 verse non-treated Huh7 cells. (D) Quantify of ISG12a mRNA in normal liver tissues and liver cancer tissues. Total cellular RNA was isolated from liver cancer tissues and normal liver tissues. ISG12a mRNA was examined by real-time PCR and normalized with GAPDH.

The intrinsic and extrinsic pathways are two major signaling pathways that lead to apoptosis in mammalian cells [24]. To investigate the possible apoptotic signaling pathway of TRAIL-induced apoptosis in human HCC and GC cells, we treated LH86, HLCZ02 and HGC-27 cells with TRAIL for 4 hours in the presence of pan-caspase inhibitor z-VAD-FMK. Z-VAD-FMK significantly blocked TRAIL-induced apoptosis of HCC and GC cells, suggesting that TRAIL induces caspase-dependent apoptosis in human HCC and GC cells (Fig. 2A). Active form of caspase-9 and released cytochrome c were observed in sensitive cells (Fig. 2B), indicating that TRAIL-induced apoptosis of HCC cells was partially in a mitochondria-dependent apoptotic pathway.

Although some cancer cells are resistant to TRAIL, combination of TRAIL with other reagents significantly potentiates TRAIL-induced apoptosis in resistant cell lines, indicating that TRAIL-mediated apoptotic pathway is intact in the resistant cells [25, 26]. So we postulate that there are other genes differ in sensitive and resistant cell lines after treatment of TRAIL. To identify differently regulated genes in response to TRAIL, we profiled gene expression mediated by TRAIL in Huh7 and LH86 cells. Interestingly, cohorts of genes induced by TRAIL are IFN-stimulated genes (ISGs) including ISG12a. Our real-time PCR results confirmed that TRAIL induced ISG12a in LH86, HLCZ02 and HGC-27 cells but had no such effect in Huh7, HLCZ01 and BGC-823 cells (Fig. 2C). Moreover, the level of endogenous ISG12a was much higher in sensitive cells than that in resistant cells (Fig. 2C). To determine the time course of ISG12a expression, we treated LH86, Huh7, HLCZ01, HLCZ02, HGC-27 and BGC-823 cells with TRAIL for different time periods, and real-time PCR was used to quantify the level of ISG12a mRNA. The data showed that ISG12a is consistently highly expressed in sensitive cells compared to that in resistant cells (Fig. 2C). We wondered if this situation occurs in vivo. To answer this question, we investigated ISG12a in liver cancer specimens. ISG12a was highly expressed in normal liver tissues and less invasive liver cancer tissues as compared with aggressive liver cancer tissues (Fig. 2D).

### ISG12a regulates the sensitivity of cancer cells to TRAIL treatment *in vitro* and *in vivo*

**Figure 3:**
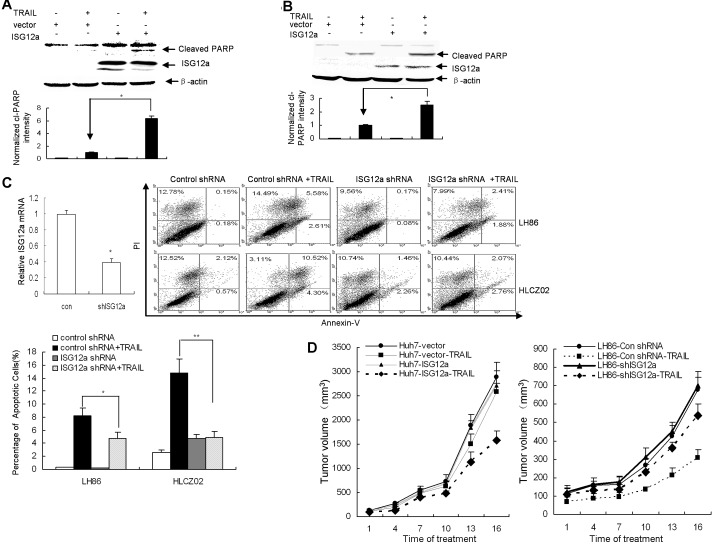
ISG12a regulates the sensitivity of cancer cells to TRAIL treatment *in vitro* and *in vivo* (A/B) Forced expression of ISG12a in TRAIL-resistant cells sensitized the cells to TRAIL treatment. Huh7 (A) or BGC-823 (B) cells were transfected by pcDNA3.1-ISG12a, followed with TRAIL treatment. ISG12a and PARP activation was detected by western blot (upper). Cleaved PARP level was quantified by densitometry and normalized to β-actin (lower). The data represented the means of 3 independent experiments. *P<0.05 verse vector-transfected cells. (C) Knockdown ISG12a renders sensitive cells resistant to TRAIL-induced apoptosis. LH86 and HLCZ02 cells were transfected by pSilencer-ISG12a shRNA or pSilencer-control shRNA, followed with TRAIL treatment for 4 hours. Silencing of ISG12a was confirmed by real-time PCR. The cells were collected and analyzed by flow cytometry. The images are one representative of three independent experiments. The results are the average of 3 independent experiments. (D) ISG12a regulates the sensitivity of cancer cells to TRAIL treatment in vivo. The detail protocol was described in “Material and Methods” section.

ISG12a is highly induced by type I IFN in many cell types and impact apoptosis [18, 19]. Moreover, TRAIL induced higher level of ISG12a in sensitive cells than in resistant cells (Fig. 2C). To assess the impact of ISG12a on apoptotic processes in TRAIL-resistant HCC cells, we delivered the plasmid pcDNA3.1-ISG12a into Huh7 cells. As expected, overexpression of ISG12a increased TRAIL-induced PARP activation as assessed by the appearance of the cleaved fragments in the cells (Fig. 3A). To further confirm the effects of ISG12a on apoptotic processes in TRAIL-resistant cells, we established a TRAIL-resistant clone of LH86 cells, LH86-TR cells, obtained by in vitro selection for TRAIL resistance (Supplementary Fig. S2A). Then we transfected pcDNA3.1-ISG12a into LH86-TR cells and treated the cells with TRAIL. Increased activation of PARP was observed in resistant cells overexpressing ISG12a (Supplementary Fig. S2B). Enhanced activation of PARP was also observed in resistant GC cells BGC-823 when the cells were transfected with pcDNA3.1-ISG12a (Fig. 3B). To ensure that ISG12a expression was responsible for sensitizing the cells to the proapoptotic stimulus TRAIL, we used short hairpin RNA (shRNA) constructs pSilence-ISG12a shRNA designed to silence ISG12a in LH86 cells (Fig. 3C). A shRNA plasmid, encoding a scrambled shRNA sequence that does not lead to the specific degradation of any known cellular mRNA, was used as control. Compared with control shRNA, transfection of ISG12a shRNA caused a corresponding loss of sensitization to TRAIL-induced apoptosis in LH86 and HLCZ02 cells (Fig. 3C). These data suggested that ISG12a regulates the sensitivity of cancer cells to TRAIL-induced apoptosis in vitro.

To test whether the high sensitivity of cancer cells for apoptosis induction can be translated in cancer treatment in vivo, we silenced ISG12a in LH86 or overexpressed ISG12a in Huh7 cells and implanted these cells into the right dorsal sides of immunodeficiency NOD/SCID mice respectively. TRAIL treatment was initiated 10 days afterwards. Silencing ISG12a in TRAIL-sensitive HCC cells conferred resistance to TRAIL-induced apoptosis over control tumor (Fig. 3D). Conversely, forced expression of ISG12a in TRAIL-resistant HCC cells sensitized the cells to TRAIL treatment (Fig. 3D). These findings suggested that ISG12a is an important target for TRAIL resistance and might play an important role in tumorigenicity of cancer cells.

### MicroRNA-942 (miR-942) directly targets 3'UTR of ISG12a

**Figure 4:**
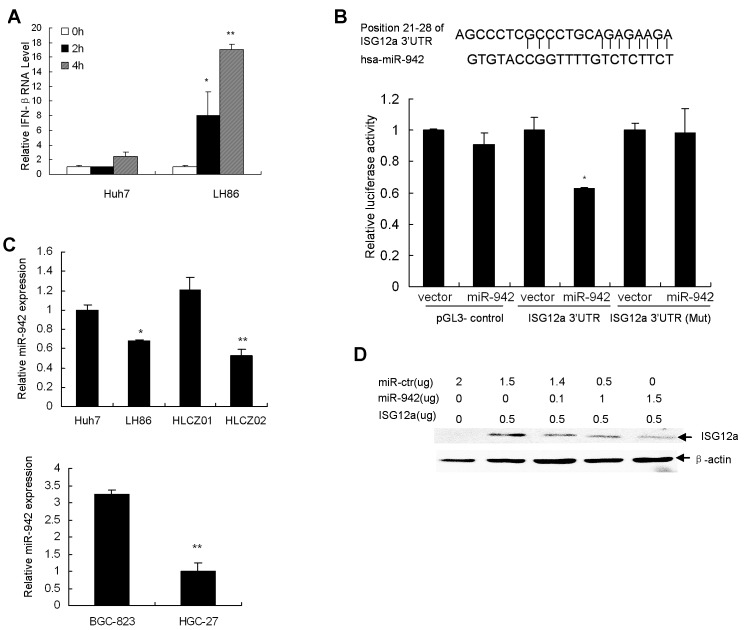
MiR-942 directly targets 3'UTR of ISG12a (A) TRAIL induced IFN-β in sensitive cells. LH86 and Huh7 cells were treated by TRAIL. IFN-β mRNA was detected by real-time PCR and normalized with GAPDH. The data represented the means of 3 different experiments. *P<0.05, **P<0.01 verse control non-treated cells. (B) ISG12a 3'UTR contains one predicted miR-942 binding site. In the figure the alignment of the seed region of miR-942 with 3'UTR of ISG12a is shown. pGL3-ISG12a-UTR luciferase construct containing wild type or mutated (Mut) ISG12a 3'UTR was transfected into CHO cells together with pcDNA3.1-miR-942. Relative repression of firefly luciferase expression was standardized to transfection control. Results are the average of 3 independent experiments performed in triplicate. *P<0.05 verse vector-treated cells. (C) miR-942 is significantly upregulated in TRAIL-resistant cells, verse TRAIL-sensitive cells. MiR-942 was examined by real-time PCR and normalized with U6. The data represented the means of three independent experiments. *P<0.05, **P<0.01 verse Huh7 cells. (D) The effect of miR-942 overexpression on ISG12a level in cancer cells. pcDNA3.1-ISG12a were transfected into LH86 cells with or without pcDNA3.1-miR-942. V5-tagged ISG12a protein was determined with V5 antibody by Western blot. A representative result of one from three independent experiments is presented.

ISG12a is highly induced by type I IFN. The stimulation of ISG12a by TRAIL led us to ascertain whether ISG12a would be induced by type I IFN in our study. We assume that TRAIL induces ISG12a through IFN signaling pathway. To further explore the mechanisms of ISG12a in TRAIL-induced apoptosis, we examined the expression of IFN-β in TRAIL-treated cancer cells. Interestingly, TRAIL indeed induced IFN-β in sensitive LH86 cells (Fig. 4A). To investigate whether IFN-β was involved in induction of ISG12a by TRAIL in sensitive cells, we used specific shRNA to silence IFN-α/β receptor (IFNR) and then evaluated the expression of ISG12a. Surprisingly, specific IFNR shRNA lead to reduction of endogenous expression of IFNR by 50% while it had no effect on the induction of ISG12a by TRAIL (Supplementary Fig. S3A). In consistent with the data, IFNR shRNA had no effect on the TRAIL sensitivity of LH86 cells (Supplementary Fig. S3B). The results indicated that induction of ISG12a by TRAIL in cancer cells is independent of IFN signaling.

MiRNAs regulate the expression of many cellular proteins and are differentially expressed in cancer cells versus normal cells. To determine the mechanisms implicated in the repression of ISG12a in resistant cells, we performed a bioinformatics search (Targetscan, Pictar, RNhybrid) the putative microRNA targeting ISG12a. Among the candidate targets, 3'UTR of human ISG12a contains region that matches the seed sequence of human miR-942 (Fig. 4B). We predicted that ISG12a is a targeted gene of miR-942. To validate this hypothesis, we cloned 3'UTR of ISG12a containing miR-942 binding sites into downstream of the luciferase open reading frame of pGL3 control vector. The reporter construct pGL3-ISG12aUTR and pcDNA3.1-miR-942 were used to transfect CHO cells, which express very low level of endogenous miR-942. Increased expression of miR-942 upon transfection significantly decreased luciferase activity measured as relative luciferase activity (Fig. 4B). Conversely, when we performed luciferase assays using a plasmid harboring ISG12a3'UTR(Mut), where the binding sites for miR-942 were inactivated by site-directed mutagenesis, we did not observe inhibitory effect of miR-942 (Fig. 4B). Moreover, miR-942 is significantly upregulated in TRAIL-resistant Huh7 and HLCZ01 cells, verse TRAIL-sensitive LH86 and HLCZ02 cells (Fig. 4C). To test whether miR-942 affects ISG12a expression, we examined the effect of miR-942 forced expression on ISG12a level in LH86 cells. Overexpression of miR-942 upon transfection markedly reduced the level of ISG12a compared to LH86 cells transfected with scrambled pre-miR (Fig. 4D). All the data suggested that the 3'UTR of ISG12a is a direct target of miR-942.

### MiR-942 is inversely correlated with ISG12a expression in cancer tissues

**Figure 5:**
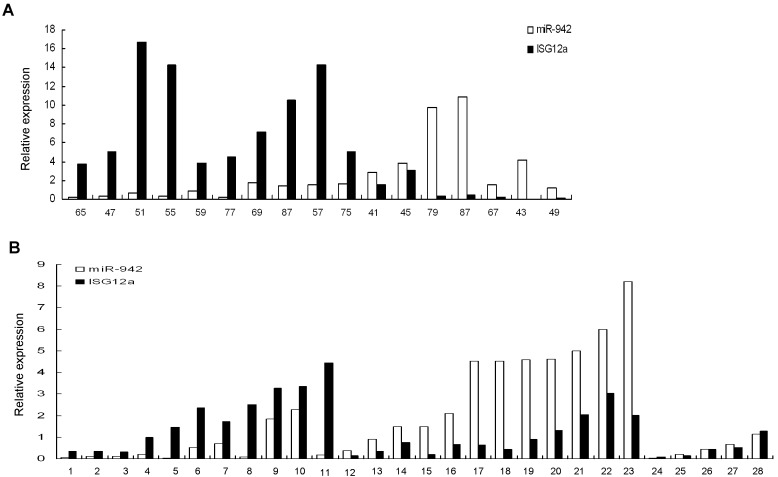
MiR-942 is inversely correlated with ISG12a expression in cancer tissues Total cellular RNA was isolated from 17 liver cancer tissues (A) or 28 gastric cancer tissues (B). MiR-942 or ISG12a was examined by real-time PCR. The association between miR-942 and ISG12a for 17 subjects in liver cancer tissues or for 28 subjects in gastric cancer tissues was calculated statistically using the Pearson correlation coefficient and the respective p value, 15 of 17 liver cancer tissues or 23 of 28 gastric cancer tissues significant at p<0.05. The pearson correlation indicated that an inverse relation between miR-942 and ISG12a in liver and gastric cancer tissues.

Our data show that ISG12a increases in less invasive liver cancer tissues and TRAIL-sensitive cells, as compared with aggressive liver cancer tissues and resistant cells respectively. MiR-942 is significantly upregulated in TRAIL-resistant cancer cells, verse sensitive cancer cells. Furthermore, we found an inverse correlation between miR-942 expression and ISG12a expression in cancer cell lines analyzed (Fig. 2C and Fig. 4C). All the data indicated that high expression of miR-942 might be one of the mechanisms acting to negatively regulate ISG12a in cancer cells. We wondered if this regulation occurs in vivo. To answer this question, we examined the expression of miR-942 and ISG12a by real-time PCR in primary liver cancer and gastric cancer tissue samples. Of the 17 primary liver cancer tissues examined, miR-942 is inversely correlated with ISG12a expression in 15 liver cancer tissues (Fig. 5A). Moreover, miR-942 is inversely correlated with ISG12a expression in 23 of 28 gastric cancer tissues (Fig. 5B). The pearson correlation indicated that an inverse relation between miR-942 and ISG12a in cancer tissues exists. The data further support the finding that ISG12a may be a target of miR-942 *in vivo*.

### MiR-942 modulates the sensitivity of cancer cells to TRAIL-induced apoptosis by targeting ISG12a

**Figure 6:**
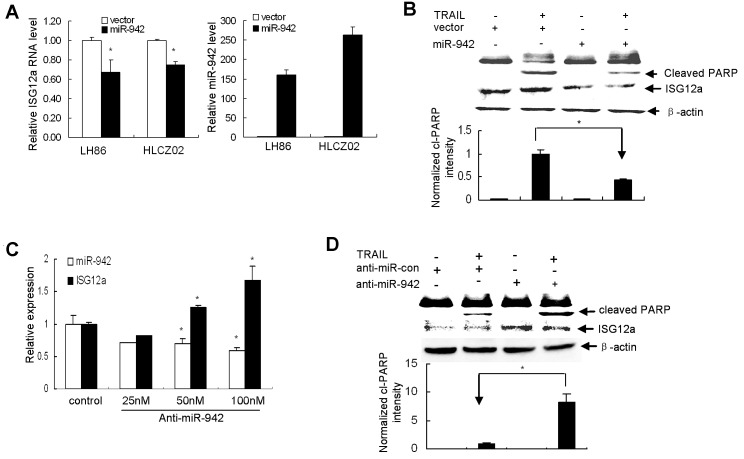
MiR-942 modulates the sensitivity of cancer cells to TRAIL-induced apoptosis by targeting ISG12a (A) Forced expression of miR-942 in LH86 or HLCZ02 cells significantly reduced the endogenous level of ISG12a. LH86 or HLCZ02 cells were transfected with pcDNA3.1-miR-942. MiR-942 or ISG12a mRNA was examined by real-time PCR and normalized with U6 or GAPDH respectively. The data represented the means of 3 independent experiments. (B) Forced expression of miR-942 in LH86 cells changed the TRAIL sensitive phenotype to a resistant one. LH86 cells were transfected with pcDNA3.1-miR-942, followed with TRAIL treatment for 4 hours. Activation of PARP and ISG12a was determined by western blot. Representative image was shown. (C) Huh7 cells were transfected by anti-miR-942. MiR-942 or ISG12a mRNA was examined by real-time PCR and normalized with U6 or GAPDH respectively. The data represented the means of at least 3 different experiments. (D) Huh7 cells were transfected by anti-miR-942, followed by TRAIL treatment for 4 hours. Activation of PARP and ISG12a was determined by western blot. Representative image was shown.

To examine the effect of miR-942 forced expression on the expression of ISG12a and TRAIL sensitivity in cancer cells, we delivered pcDNA3.1-miR-942 into LH86 or HLCZ02 cells. Interestingly, forced expression of miR-942 in LH86 or HLCZ02 cells significantly reduced the endogenous level of ISG12a (Fig. 6A) and changed the TRAIL sensitive phenotype to a resistant one, as the activation of PARP evidenced by appearance of cleaved PARP fragment was impaired (Fig. 6B). Conversely, knockdown of miR-942 expression by anti-miR-942 in Huh7 cells, which was confirmed by real-time PCR, restored the expression of ISG12a in resistant cells (Fig. 6C) and sensitized the cells to TRAIL-induced apoptosis (Fig. 6D). To further corroborate the negative regulation of miR-942 on TRAIL sensitivity in cancer cells, we delivered pcDNA3.1-miR-942 into HGC-27 cells. Forced expression of miR-942 in sensitive gastric cancer cell HGC-27 significantly reduced the endogenous level of ISG12a and changed the TRAIL sensitive phenotype to a resistant one (Supplementary Fig. S4A). Conversely, knockdown of miR-942 expression by anti-miR-942 restored the expression of ISG12a in resistant BGC-823 cells and sensitized the cells to TRAIL-induced apoptosis (Supplementary Fig. S4B). The expression of TRAIL receptors is not affected by miR-942 (data not shown). All together, these data suggested that miR-942 modulates sensitivity of cancer cells to TRAIL-mediated apoptosis by targeting ISG12a.

### AKT controls TRAIL resistance of cancer cells through downregulation of ISG12a by miR-942

**Figure 7:**
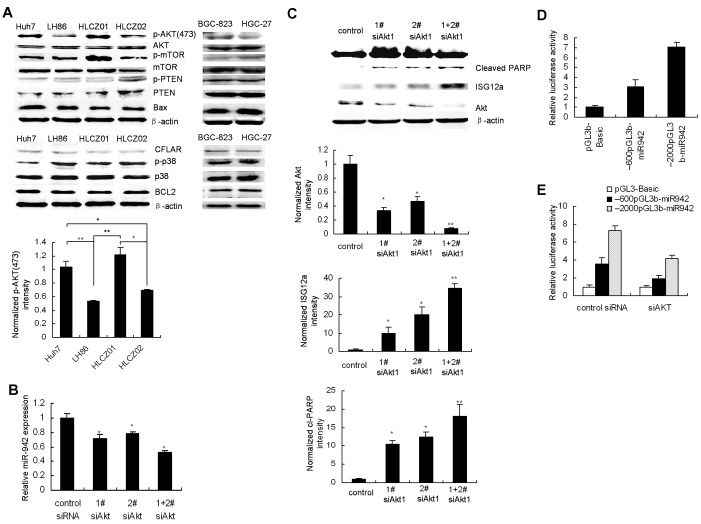
AKT controls TRAIL resistance of cancer cells through downregulation of ISG12a by miR-942 (A) Activation of Akt in TRAIL-resistant HCC and gastric cancer cells. Protein was purified form Huh7, LH86, HLCZ01, HLCZ02, HGC-27 and BGC-823 cells. AKT, p-AKT, PTEN, mTOR, p38, Bax, and Bcl2 protein were examined by western blot analysis. (B) SiRNAs targeting AKT were transfected into Huh7 cells. The expression of miR-942 in the cells was detected by real-time PCR and normalized to U6. (C) SiRNAs targeting AKT were transfected into Huh7 cells. The cells were treated by TRAIL for 4 hours. The expression of AKT, ISG12a, and cleaved PARP was evaluated by western blot analysis. Representative image was shown. The level of AKT, ISG12a or cleaved PARP, was quantified by densitometry and normalized to β-actin respectively. Representative image was shown. Results are the average of three independent experiments. *P<0.05, **P<0.01 verse control siRNA-treated cells. (D) Luciferase assays were carried out to identify the miR-942 promoter, (E) AKT regulation on miR-942 expression.

Activation of AKT signaling is a frequent event observed in many types of cancers, including gastric and liver cancer [27, 28]. AKT activation was observed in TRAIL-resistant cancer cells compared with sensitive cells (Fig. 7A) while there was no marked difference of other survival factors including such as mTOR, CFLAR(FLIP) and Bcl2, pro-apoptotic factor such as BAX, tumor suppressor PTEN and p38 in TRAIL-sensitive and resistant cancer cells (Fig. 7A). Furthermore, one recent study showed that miR-21 is positively regulated via an AKT-dependent pathway [29]. We postulate that AKT may regulate miR-942 expression. When we used AKT inhibitor to block AKT activation, miR-942 was downregulated and ISG12a was upregulated (Supplementary Fig. S5A-C). To further investigate whether miR-942 is regulated by AKT, we silenced AKT in Huh7 cells. First, after AKT knockdown, miR-942 expression was downregulated (Fig. 7B). AKT knockdown was confirmed (Fig. 7C). Second, by immunostaining, we observed increased ISG12a expression levels after AKT downregulation (Fig. 7C). Meanwhile, downregulation of AKT leads to restoration of the TRAIL-induced apoptosis (Fig. 7C). Overexpression of miR-942 or silencing ISG12a reversed TRAIL-induced apoptosis by AKT knockdown (Supplementary Fig.S6). These data collectively suggested that AKT controls TRAIL resistance of cancer cells through downregulation of ISG12a by miR-942.

To elucidate the direct involvement of AKT on miR-942 expression levels, we analyzed the upstream sequence of miR-942 with the Promoter.2 prediction server. We found two DNA fragments containing the putative regulatory region upstream to miR-942(from +1~-600nt, +1~-2000nt). The two regions were amplified and cloned into pGL3basic. The luciferase assay showed that both sequences increased luciferase activity compared with the vector suggesting that both regions could be the promoter of miR-942 (Fig. 7D). To confirm that the two regulatory regions were regulated by AKT, we knocked down AKT by using siRNA and found decreased luciferase activity after two reporter constructs overexpression (Fig. 7E). These results indicated that miR-942 promoting sequences are regulated by AKT.

## DISCUSSION

HCC and gastric cancer are two of the most common causes of cancer-related death worldwide. The prognosis is poor. One of the important factors affecting survival rate is resistance to therapeutic agents. Owing to its specific toxicity for cancer cells, recombinant TRAIL is among the most promising apoptosis-based antitumor agents. Therapy based on TRAIL is now in phase 2 clinical trial in different types of cancers. However, many cancer cells remain resistant to TRAIL-induced apoptosis and the mechanism of such resistance is unclear.

To identify specific signatures as potential therapeutic targets for TRAIL-resistant phenotype in human cancer, we analyzed the gene expression profile in TRIAIL-sensitive cancer cells verse resistant cancer cells. ISG12a overexpressed in tumors of epithelial origin, such as ovarian carcinoma, sclerosing basal cell carcinoma and squamous cell carcinoma [30]. Our results showed that ISG12a decreased in hepatocellular carcinoma and gastric cancer tissues compared with adjacent nontumorous tissues and it was consistently highly expressed in sensitive cells compared to that in resistant cells. These findings indicated that ISG12a may play roles in different tissues. Consistent with the recently suggested pro-apoptotic role of ISG12a [19, 31], our results showed that force expression of ISG12a in TRAIL-resistant cells restored the sensitivity in resistant cells and repression of ISG12a is implicated in causing TRAIL resistance.

MiRNAs are attractive potential drug targets since they regulate many cellular proteins and are differentially expressed in malignant cells. They may modulate TRAIL-induced apoptosis pathway. A recent study showed that miR-221&222 overexpresses in aggressive hepatocarcinoma cells regulate TRAIL resistance and enhance tumorigenicity through PTEN and TIMP3 downregulation [32]. MiR-494 modulates TRAIL-induced apoptosis through BIM down-regulation and is regulated by ERK1/2 [33]. Our data demonstrated that miR-942 is significantly upregulated in TRAIL-resistant cells or aggressive cancer tissues, verse TRAIL-sensitive cells or less invasive cancer tissue, clearly indicating that miR-942 overexpression is a prerequisite of TRAIL-resistant HCC and gastric cancer cells. In vitro and in vivo experiments revealed that elevated level of miR-942 in TRAIL-resistant cells or aggressive cancer tissues correlates with ISG12a downregulation, suggesting that miR-942 might be a causal factor in the downregulation of ISG12a in cancers. In the current study, the data indicated that miR-942 modulates TRAIL sensitivity in cancer cells mainly by targeting ISG12a. However, it seems plausible that silencing of other targets of miR-942 contributes to TRAIL resistance in cancer cells. MiR-942 may offer a novel TRAIL response marker for cancer patient selection and personalized medicine with important implications in designing new therapeutics for TRAIL resistant tumors.

ISG12a is an important antiviral factor and sensitizes cells to apoptotic stimuli [34]. One recent study implicates ISG12a as a contributing regulator of IFN-induced apoptosis, which in turn augment or support the antiviral activities of type I IFN [25]. Our data demonstrated that induction of ISG12a in cancer cells does not require IFN feedback although TRAIL induces IFN-β and ISG12a. Downregulation of ISG12a by miR-942 is needed to maintain the TRAIL-resistant phenotype of cancer cells and favors cancer cell survival, which might be the cell defense response to external harmful stimulation.

PI3K/AKT pathway is a major cell survival pathway, playing a key role in the development of multiple drug resistance, such as TRAIL [35]. There are some miRNAs which confer metastatic potentials in HCC cells or inhibit tumor growth regulated through PI3K/AKT pathway [36, 37]. We found that decreased expression level of miR-942 correlates with AKT knockdown and ISG12a expression is restored after AKT downregulation. Our data suggested that AKT controls TRAIL resistance of cancer cells through downregulation of ISG12a by miR-942. Based on our findings, TRAIL combination with AKT inhibitors may be used to counter drug resistance for cancer treatment. Taken together, all the data indicate that the intracellular miR-942 may modulate sensitivity of cancer cells to TRAIL through targeting ISG12a with important implications in the design of new therapeutic agents.

## MATERIALS and METHODS

### Cell lines

Human HCC cell lines Huh7, LH86 [20], HLCZ01 [21] and HLCZ02 were grown in Dulbecco's Modified Eagle's Medium (DMEM) with 10% fetal bovine serum at 37 °C in 5% CO_2_.

### Isolation and establishment of a novel hepatoma cell line HLCZ02

Cells were isolated from the liver tumor tissue of a male patient. Experimental procedures were performed in accordance with the Ethics Committee of Hunan Provincial Tumor Hospital. The cells were isolated and cultured as described previously [20, 21].

### Reagents and antibodies

Mouse anti-caspase-9, Bcl2, Bax, AKT, p-AKT and anti-PARP antibodies were from Cell Signaling Technology. mTOR, p- mTOR, PTEN, p-PTEN, p38, p-p38, CFLAR were from SANTA CRUZ. DR4 and DR5 were from Boster. Rabbit anti ISG12a antibody was from Abcam (ab171919). Mouse anti-β-actin and anti-V5 monoclonal antibody were from Sigma and Invitrogen respectively. Pancaspase inhibitor z-VAD-FMK was obtained from Promega. 2'-O-me-anti-miR-942 (5'-CACAUGGCCAAAACAGAGAAGA-3') & 2'-O-me-GFP miR (5'-AAGGCAAGCUGACCCUGAAGU-3') were from Takara. The Annexin V-FITC apoptosis detection kit and TRAIL were purchased from BD Pharmingen and R&D Systems respectively.

### Cancer tissue samples

A total of 17 snap-frozen normal and liver cancer tissues, 28 normal and gastric cancer tissues were collected at Hunan Provincial Tumor Hospital (Changsha, China). The human tissues were obtained and studied in strict adherence to the protocol approved by Hunan Provincial Tumor Hospital Review Board.

### Plasmid construction

For construction of the expression plasmid for ISG12a, the entire open reading frame of human ISG12a gene was polymerase chain reaction (PCR) amplified from p3XFLAG-CMV-14-ISG12a (kindly provided by Dr. Douglas W Leaman, The University of Toledo), and inserted into pcDNA3.1/V5-His (Invitrogen). The primers for amplification of ISG12a are 5'-CGCGCGGATCCATGG AGGCCTCTGCTCTC-3'(F), and 5'-CTGCAGGAATTCGTAGAACCTCGCAATGA-3'(R). The oligonucleotides encode 19-mer hairpin sequence specific to the ISG12a mRNA were incorporated into the pSilencer-neo plasmid (Ambion). The sequences of ISG12a shRNAs targeting two regions of ISG12a were 5'-AAGTTCATCCTGGGCTCCATT-3' and 5'-AATTAACCCGAGCAGGCATGG-3'. Precursor of miR-942 was amplified from Huh7 cells and inserted into pcDNA3.1/V5-His. The primers for miR-942 are 5'-GCATGGATCCGCTTTAACA ATGGTTCCTCCG-3'(F) and 5'-GCCGGTCTAGAAGCACCTTTTGTTTCTATTAT CACG-3'(R). All constructs were confirmed, and transfected into cells using Lipofectamine 2000 reagents (Invitrogen).

### DNA ladder assay

Cells were exposed to TRAIL for 4 hours. Cellular DNA was isolated and 2 μg of DNA was separated on 1% agarose gel at 50V for one hour.

### Western blot analysis

The protocol has been reported previously [21].

### Quantitative real-time PCR

The procedure has been published previously [22, 23]. The cDNA of miR-942 were synthesized from total RNA using stem-loop RT primer (5'-GTCGTATCCAGTGCAGGGTCCGAGGTATT CGCACTGGATACGACCACAT-3'), and miR-942 was quantized by real-time PCR using primers 5-GCGCGCTCTTCTCTGTTTTGGC-3' and 5'-GTGCAGGGTCCGAGGT-3'. The internal control was U6. The cDNA of U6 were synthesized from total RNA using stem-loop RT primer (5'-CGCTTCACGAATTTGCGTGTCAT-3'), and U6 was quantized by real-time PCR using primers 5'-GCTTCGGCAGCACATATACAAAAT-3' and 5'-CGCTTCACGAATTTGCGTGTCAT-3'. Fold variations were calculated after normalization to U6. ISG12 was quantized by real-time PCR using primers 5'-TGCCATGGGCTTCACTGCGG-3' and 5'-CTGCCCGAGGCAACTCCACC-3'.

### Flow cytometry analysis

The procedure has been published previously [20].

### Luciferase Assay

The 3'UTR of the human ISG12a gene were PCR amplified using the following primers: 5'-TTAATAATCTAGACTCCCTGCCCCTCGCCCTGCA-3' (F) and 5'-GCGCCGGGTCTAGAGAAGAGTTGCAACAATTCATC-3'(R). They were then cloned downstream of the Renilla luciferase stop codon in pGL3 control vector (Promega). CHO cells were cotransfected with 1μg of ISG12a-3'UTR or 1μg of ISG12a-3'-UTR-Mut plasmids and 100 ng of Renilla luciferase expression construct, pRL-CMV (Promega) using Lipofectamine 2000 (Invitrogen). Cells were harvested 24 hr posttransfection and assayed with Dual Luciferase Assay (Promega) according to the manufacturer's instructions. Three independent experiments were performed in triplicate. DNA fragments containing the putative regulatory regions upstream to miR-942 (from +1~-600 nt, +1~-2000 (+1 position corresponds to the 5' terminus of miR-942 hairpin) were amplified and cloned in pGL3basic (Promega). CHO cells were transfected with Lipofectamine 2000 (Invitrogen), 1.0 μg of pGL3basic empty vector or of pGL3 containing the above genomic fragments, 100 ng of Renilla luciferase expression construct pRL-CMV (Promega) and AKT siRNA. After 48h, cells were lysed and assayed with Dual Luciferase Assay (Promega) according to the manufacturer's instructions. The primers utilized for the cloning were the followings: −2000pGL3b Forw: 5'-ggtaccgctttagactcaataatttaggacgattag -3'

-600pGL3b Forw: 5'-ggtaccaccaggcacatcagtgtctctgttctattg -3'

miR-942 pGL3b Rev: 5'-ctcgaggtttgctgaagaagaaagtgaaag-3'

### *In vivo* animal experiments

Animal studies were performed according to the protocol approved by Hunan Provincial Tumor Hospital Review Board. Huh7 cells were transfected with pcDNA3.1-ISG12a or pcDNA3.1 vector. The plasmid pSilencer-ISG12a shRNA or pSilencer-control shRNA was delivered into LH86 cells. After selection in antibiotics for 10 days, 5 × 10^6^ cells in 200μL PBS were injected subcutaneously into the low right flank of 6-week-old male NOD/SCID immunodeficiency mice. Treatment started 10 days from the cells inoculation via tail vein injections of TRAIL (10mg/kg/day) or vehicle (PBS) on days at 1, 3, 5, 7, 9, 11, 13, 15, and 17. For all models, tumor size on two axes was measured every 3 days. The values were transformed into tumor size using the following formula: tumor volume=0.5 × width^2^ × length. Twenty-one days after injection, mice were sacrificed.

Statistical Analysis. Statistical analyses were performed with the two-tailed Student's t-test, and error bars represent S.D. *P<0.05, **P<0.01, ***P<0.001.

### COMPETING INTERESTS

The authors declare that they have no competing interests.

### AUTHER'S CONTRIBUTION

HZ conceived and designed the experiments. NL carried out the main experiments and analyzed the data. CZ collected tissue samples. NL and XW established the cell lines. NL, TC and DY did the animal experiments. WJ helped design the experiments. HZ wrote the paper. All authors read and approved the final manuscript.

## SUPPLEMENTARY FIGURES



## References

[1] Jemal A, Bray F, Center MM, Ferlay J, Ward E, Forman D (2011). Global cancer statistics. CA Cancer J Clin.

[2] Kane RC, Farrell AT, Saber H, Tang S, Williams G, Jee JM, Liang C, Booth B, Chidambaram N, Morse D, Sridhara R, Garvey P, Justice R, Pazdur R (2006). Sorafenib for the treatment of advanced renal cell carcinoma. Clin Cancer Res.

[3] Lang L (2008). FDA approves sorafenib for patients with inoperable liver cancer. Gastroenterology.

[4] Llovet JM, Ricci S, Mazzaferro V, Hilgard P, Gane E, Blanc JF, de Oliveira AC, Santoro A, Raoul JL, Forner A, Schwartz M, Porta C, Zeuzem S, Bolondi L, Greten TF, Galle PR (2008). SHARP Investigators Study Group. Sorafenib in advanced hepatocellular carcinoma. New Engl J Med.

[5] Wang S (2008). The promise of cancer therapeutics targeting the TNF-related apoptosis-inducing ligand and TRAIL receptor pathway. Oncogene.

[6] Mahmood Z, Shukla Y (2010). Death receptors: targets for cancer therapy. Exp Cell Res.

[7] Ellen JE, EI-Deiry WS (2012). Regulation of human TRAIL gene. Cancer Biol Ther.

[8] Micheau O, Shirley S, Dufour F (2013). Death receptors as targets in cancer. Br J Pharmacol.

[9] Ashkenazi A (2002). Targeting death and decoy receptors of the tumor-necrosis factor superfamily. Nat Rev Cancer.

[10] Ashkenazi A, Dixit V (1998). Death receptors: signaling and modulation. Science.

[11] Johnstone RW, Frew AJ, Smyth M (2008). The TRAIL apoptotic pathway in cancer onset, progression and therapy. Nat Rev Cancer.

[12] Gerspach J, Pfizenmaier K, Wajant H (2011). The therapeutic targeting of CD95 and the TRAIL death receptors. Recent Pat Anticancer Drug Discov.

[13] Stagg J, Sharkey J, Pommey S, Young R, Takeda K, Yagita H, Johnstone RW, Smyth MJ (2008). Antibodies targeted to TRAIL receptor-2 and ErbB-2 synergize in vivo and induce an antitumor immune response. Proc Natl Acad Sci USA.

[14] Hall MA, Cleveland JL (2007). Clearing the TRAIL for cancer therapy. Cancer Cell.

[15] Mahalingam D, Szegezdi E, Keane M, de Jong S, Samali A (2009). TRAIL receptor signaling and modulation: Are we on the right TRAIL?. Cancer Treat Rev.

[16] Bansal H, Seifert T, Bachier C, Rao M, Tomlinson G, Iyer SP, Bansal S (2012). The transcription factor Wilms tumor 1 confers resistance in myeloid leukemia cells against the proapoptotic therapeutic agent TRAIL (tumor necrosis factor α-related apoptosis-inducing ligand) by regulating the antiapoptotic protein Bcl-xL. J Biol Chem.

[17] Hong S, Kim HY, Kim J, Ha HT, Kim YM, Bae E, Kim TH, Lee KC, Kim SJ (2013). Smad7 protein induces interferon regulatory factor 1-dependent transcriptional activation of caspase 8 to restore tumor necrosis factor-related apoptosis-inducing ligand (TRAIL)-mediated apoptosis. J Biol Chem.

[18] Malhotra S, Bustamante MF, Pérez-Miralles F, Rio J, Ruiz de Villa MC, Vegas E, Nonell L, Deisenhammer F, Fissolo N, Nurtdinov RN, Montalban X, Comabella M (2011). Search for specific biomarkers of IFN β bioactivity in patients with multiple sclerosis. PLoS One.

[19] Rosebeck S, Leaman DW (2008). Mitochondrial localization and pro-apoptotic effects of the interferon-inducible protein ISG12a. Apoptosis.

[20] Zhu H, Dong H, Eksioglu E, Hemming A, Cao M, Crawford JM, Nelson DR, Liu C (2007). Hepatitis C virus triggers cell death through innate intracellular antiviral defense system. Gastroenterology.

[21] Yang D, Zuo C, Wang X, Meng X, Xue B, Liu N, Yu R, Qin Y, Gao Y, Wang Q, Hu J, Wang L, Zhou Z, Tan D, Guang Y, Zhu H (2014). Complete replication of hepatitis B virus and hepatitis C virus in a newly developed hepatoma cell line. Proc Natl Acad Sci USA.

[22] Shi S, Yu X, Gao Y, Xue B, Wu X, Wang X, Yang D, Zhu H (2014). Inhibition of hepatitis C virus production by aptamer for core protein. J Virol.

[23] Yang D, Meng X, Yu Q, Xu L, Long Y, Liu B, Fang X, Zhu H (2013). Inhibition of hepatitis C virus infection by DNA aptamer against envelop protein. Antimicrob Agents and Chemother.

[24] Johnstone RW, Frew AJ, Smyth MJ (2008). The TRAIL apoptotic pathway in cancer onset, progression and therapy. Nat Rev Cancer.

[25] Chen KF, Tai WT, Liu TH, Huang HP, Lin YC, Shiau CW (2010). Sorafenib overcomes TRAIL resistance of hepatocellular carcinoma cells through the Inhibition of STAT3. Clin Cancer Res.

[26] Wang W, Gallant JN, Katz SI, Dolloff NG, Smith CD, Abdulghani J, Li PK, Chen PJ, Cheng AL (2011). Quinacrine sensitizes hepatocellular carcinoma cells to TRAIL and chemotherapeutic agents. Cancer Biol Ther;.

[27] Calvisi DF, Wang C, Ho C, Ladu S, Lee SA, Mattu S, Destefanis G, Delogu S, Zimmermann A, Ericsson J, Brozzetti S, Staniscia T, Chen X, Dombrowski F, Evert M (2011). Increased lipogenesis, induced by AKT-mTORC1-RPS6 signaling, promotes development of human hepatocellular carcinoma. Gastroenterology.

[28] Yoo YA, Kang MH, Lee HJ, Kim BH, Park JK, Kim HK, Kim JS, Oh SC (2011). Sonic hedgehog pathway promotes metastasis and lymphangiogenesis via activation of Akt, EMT, and MMP-9 pathway in gastric cancer. Cancer Res.

[29] Sayed D, He M, Hong C, Gao S, Rane S, Yang Z, Abdellatif M (2010). MicroRNA-21 is a downstream effector of AKT that mediates its antiapoptotic effects via suppression of Fas ligand. J Biol Chem.

[30] Martensen PM, Justesen J (2004). Small ISGs coming forward. J Interferon Cytokine Res.

[31] Makovitzki-Avraham E, Daniel-Carmi V, Alteber Z, Farago M, Tzehoval E, Eisenbach L (2013). The human ISG12a gene is a novel caspase dependent and p53 independent pro-apoptotic gene that is overexpressed in breast cancer. Cell Biology International Reports.

[32] Garofalo M, Di Leva G, Romano G, Nuovo G, Suh SS, Ngankeu A, Taccioli C, Pichiorri F, Alder H, Secchiero P, Gasparini P, Gonelli A, Costinean S, Acunzo M, Condorelli G, Croce CM (2009). miR-221&222 Regulate TRAIL Resistance and Enhance Tumorigenicity through PTEN and TIMP3 Downregulation. Cancer cell.

[33] Romano G, Acunzo M, Garofalo M, Di Leva G, Cascione L, Zanca C, Brad B, Gerolama C, Croce CM (2012). MiR-494 is regulated by ERK1/2 and modulates TRAIL-induced apoptosis in non–small-cell lung cancer through BIM down-regulation. Proc Natl Acad Sci USA.

[34] Cheriyath V, Leaman DW, Borden EC (2011). Emerging roles of FAM14 family members in innate immunity and cancer. J Inter Cytok Res.

[35] Opel D, Naumann I, Schneider M, Bertele D, Debatin KM, Fulda S (2011). Targeting aberrant PI3K/Akt activation by PI103 restores sensitivity to TRAIL-induced apoptosis in neuroblastoma. Clin Cancer Res.

[36] Wong QW, Ching AK, Chan AW, Choy KW, To KF, Lai PB, Wong N (2010). MiR-222 overexpression confers cell migratory advantages in hepatocellular carcinoma through enhancing AKT signaling. Clin Cancer Res.

[37] Fang Y, Xue JL, Shen Q, Chen J, Tian L (2012). MicroRNA-7 inhibits tumor growth and metastasis by targeting the phosphoinositide3-kinase/Akt pathway in hepatocellular carcinoma. Hepatology.

