# Supplementary material for: miR-942 decreases TRAIL-induced apoptosis through ISG12a downregulation and is regulated by AKT
Source: Oncotarget. 2014 Jun 6;5(13):4959–71. doi: 10.18632/oncotarget.2067 (PMC4148114; doi:10.18632/oncotarget.2067)
Supplement: Supplementary file 1 [file oncotarget-05-4959-s001.pdf]

# miR-942 decreases TRAIL-induced apoptosis through ISG12a downregulation and is regulated by AKT

## Supplementary Material

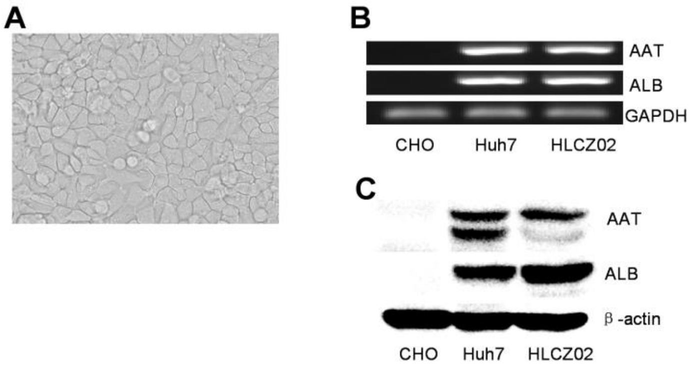


**Figure S1: Establishment of HLCZ02 cells. (A)** Morphology of HLCZ02 cell line derived from HCC tissue. **(B)** HLCZ02 cells express liver-specific genes α1-antitrypsin (AAT) and albumin. Total cellular RNA was isolated from CHO, Huh7 and HLCZ02 cells. AAT and albumin mRNA was detected by RT-PCR. CHO and Huh7 were used as negative and positive control respectively. **(C)** HLCZ02 cells express liver-specific proteins AAT and albumin. Protein was isolated from CHO, Huh7 and HLCZ02 cells. AAT and albumin protein was detected by western blot.

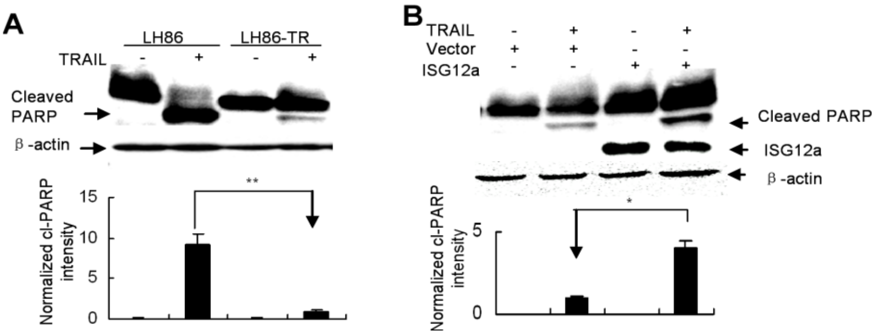


**Figure S2: ISG12a regulates the sensitivity of liver cancer cells to TRAIL treatment in vitro (A)** Establishment of acquired TRAIL-resistant LH86-TR cell by in vitro selection for TRAIL resistance. LH86 and LH86-TR cells were treated by TRAIL for 4 hours. Protein was purified and PARP activation was examined by western blot (upper). Cleaved PARP level was quantified by densitometry and normalized to β-actin (lower). The data represented the means of 3 independent experiments. ***P*<0.01. **(B)** Forced expression of ISG12a enhanced TRAIL toxicity to resistant LH86-TR. We transfected pcDNA3.1-ISG12a into LH86-TR cells and treated the cells with TRAIL for 4 hours. PARP activation and ISG12a was examined by western blot (upper panel). Cleaved PARP level was quantified by densitometry and normalized to β-actin (lower panel). A representative result of one from three independent experiments is presented. **P*<0.05 verse vector-transfected cells.

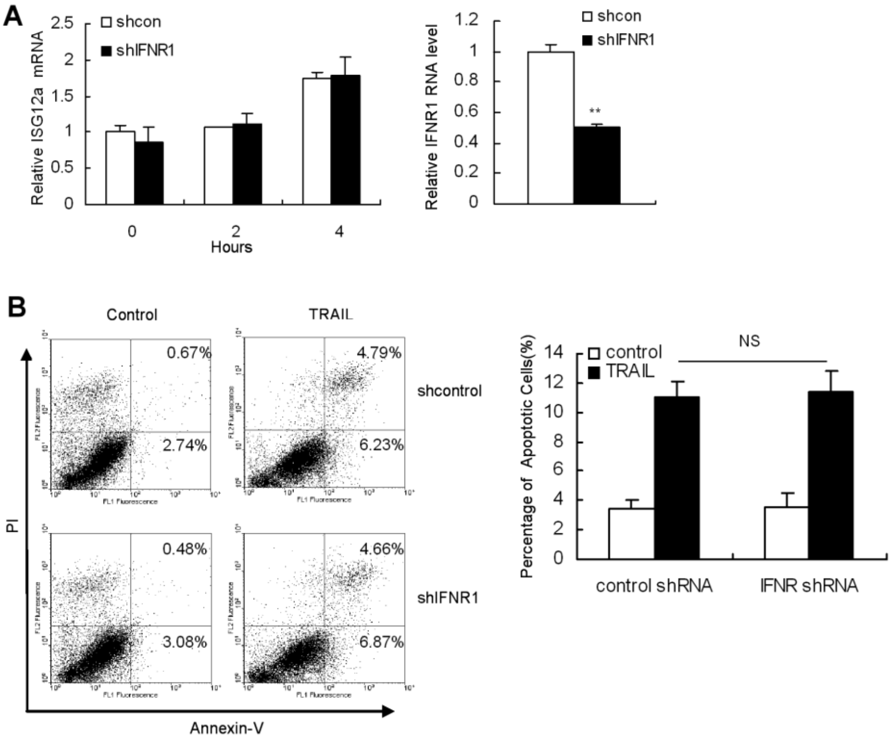


**Figure S3: Induction of ISG12a in HCC cells not through TRAIL-mediated IFN signaling. (A)** The effect of silencing IFNR1 on the induction of ISG12a by TRAIL. LH86 cells were transfected by IFNR1 shRNA, followed with TRAIL treatment for 4 hours. ISG12a (left) and IFNR1 (right) mRNA was detected by real-time PCR and normalized with GAPDH. The data represented the means of three independent experiments. **(B)** IFNR shRNA had no effect on the TRAIL sensitivity of cancer cells. LH86 cells were transfected by IFNR1 shRNA, followed with TRAIL treatment for 4 hours. The cells were examined by flow cytometry. A representative result of one from three independent experiments is presented. NS, not significant.

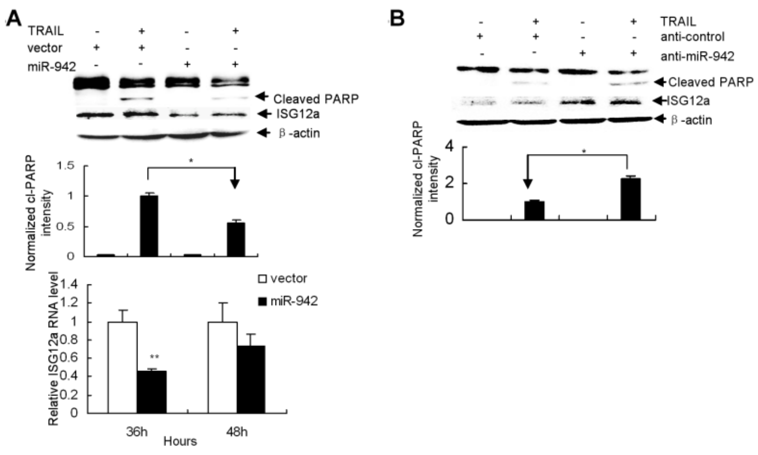


**Figure S4: MiR-942 modulates the sensitivity of gastric cancer cells to TRAIL-induced apoptosis by targeting ISG12a. (A)** Forced expression of miR-942 in gastric cancer cell HGC-27 changed the TRAIL sensitive phenotype to a resistant one. HGC-27 cells were transfected with pcDNA3.1-miR-942, followed with TRAIL treatment for 4 hours. The activation of PARP and ISG12a was determined by western blot. Representative image was shown (upper). Cleaved PARP level was quantified by densitometry and normalized to β-actin (middle). ISG12a mRNA was examined by real-time PCR (lower). The data represented the means of 3 independent experiments. **(B)** Knockdown of miR-942 by anti-miR-942 increased the level of ISG12a in TRAIL-resistant BGC-823 cells and sensitized the cells to TRAIL-induced apoptosis. BGC-823 cells were treated with anti-miR-942. ISG12a protein and the activation of PARP were determined by western blot. Representative image was shown. Cleaved PARP level was quantified by densitometry and normalized to β-actin. A representative result of one from three independent experiments is presented. *$P$<0.05 verse control cells.

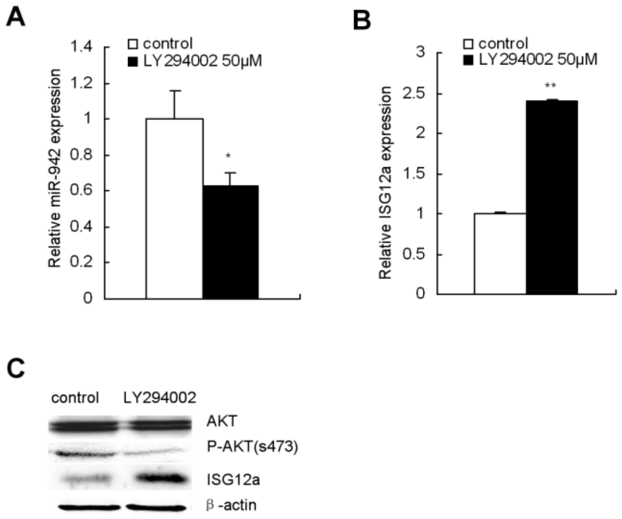


**Figure S5: Effect of AKT inhibitor on the expression of miR-942 and ISG12a in resistant cells.** Huh7 cells were treated by AKT inhibitor LY294002 for 4 hours. The expression of miR-942 **(A)** and ISG12a **(B)** was detected by real-time PCR and normalized with U6 and GAPDH respectively. The data represented the means of 3 independent experiments. **(C)** ISG12a and p-AKT protein was detected by western blot. *$P$<0.05, **$P$<0.01 verse non-treated cells.

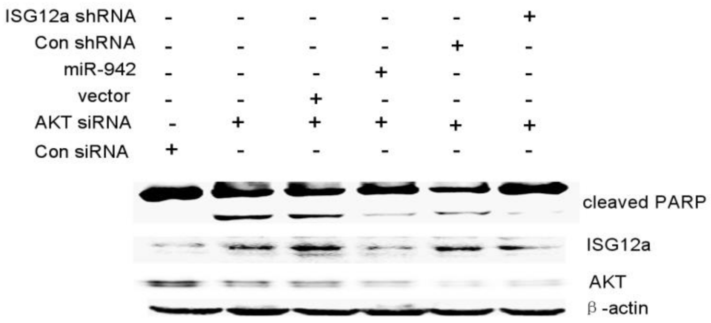


**Figure S6: Overexpression of miR-942 or silencing ISG12a reversed TRAIL-induced apoptosis by AKT knockdown.** AKT siRNA or control siRNA was transfected into Huh7 cells. Then pcDNA3.1-miR-942 or pSilencer-ISG12a shRNA was delivered into the cells, followed with TRAIL treatment for 4 hours. PARP, ISG12a and AKT were detected by western blot analysis.
